# Proteomics and Phosphoproteomics of Circulating Extracellular Vesicles Provide New Insights into Diabetes Pathobiology

**DOI:** 10.3390/ijms23105779

**Published:** 2022-05-21

**Authors:** Yury O. Nunez Lopez, Anton Iliuk, Alejandra M. Petrilli, Carley Glass, Anna Casu, Richard E. Pratley

**Affiliations:** 1 AdventHealth, Translational Research Institute (TRI), Orlando, FL 32828, USA; Yury.NunezLopez@AdventHealth.com (Y.O.N.L.); Alejandra.Petrilli@AdventHealth.com (A.M.P.); Carley.Glass@AdventHealth.com (C.G.); Anna.Casu@AdventHealth.com (A.C.); 2 Tymora Analytical Operations, West Lafayette, IN 47906, USA

**Keywords:** extracellular vesicle, exosome, multi-omics, proteomics, phosphoproteomics, prediabetes, type 2 diabetes, human

## Abstract

The purpose of this study was to define the proteomic and phosphoproteomic landscape of circulating extracellular vesicles (EVs) in people with normal glucose tolerance (NGT), prediabetes (PDM), and diabetes (T2DM). Archived serum samples from 30 human subjects (*n* = 10 per group, ORIGINS study, NCT02226640) were used. EVs were isolated using EVtrap^®^. Mass spectrometry-based methods were used to detect the global EV proteome and phosphoproteome. Differentially expressed features, correlation, enriched pathways, and enriched tissue-specific protein sets were identified using custom R scripts. Phosphosite-centric analyses were conducted using directPA and PhosR software packages. A total of 2372 unique EV proteins and 716 unique EV phosphoproteins were identified among all samples. Unsupervised clustering of the differentially expressed (fold change ≥ 2, *p* < 0.05, FDR < 0.05) proteins and, particularly, phosphoproteins showed excellent discrimination among the three groups. CDK1 and PKCδ appear to drive key upstream phosphorylation events that define the phosphoproteomic signatures of PDM and T2DM. Circulating EVs from people with diabetes carry increased levels of specific phosphorylated kinases (i.e., AKT1, GSK3B, LYN, MAP2K2, MYLK, and PRKCD) and could potentially distribute activated kinases systemically. Among characteristic changes in the PDM and T2DM EVs, “integrin switching” appeared to be a central feature. Proteins involved in oxidative phosphorylation (OXPHOS), known to be reduced in various tissues in diabetes, were significantly increased in EVs from PDM and T2DM, which suggests that an abnormally elevated EV-mediated secretion of OXPHOS components may underlie the development of diabetes. A highly enriched signature of liver-specific markers among the downregulated EV proteins and phosphoproteins in both PDM and T2DM groups was also detected. This suggests that an alteration in liver EV composition and/or secretion may occur early in prediabetes. This study identified EV proteomic and phosphoproteomic signatures in people with prediabetes and T2DM and provides novel insight into the pathobiology of diabetes.

## 1. Introduction

Type 2 diabetes mellitus (T2DM) affects 31 million people in the United States and 463 million globally [1], with a high risk for chronic complications of cardiovascular disease, chronic kidney disease, and heart failure [2]. T2DM can be prevented with lifestyle interventions and pharmacologic therapies targeting those at high risk of progressing [3,4]. These interventions are not effectively employed, however, suggesting a need to personalize therapies. There is now evidence that classic T2DM is, in fact, genetically and phenotypically heterogeneous [5]. Thus, although a large number of therapies are available to improve glucose levels in T2DM, there is a need for better biomarkers to select optimal therapies to improve outcomes and decrease morbidity, mortality, and costs from diabetes.

Although suitable progress has been achieved in the development of biomarkers with proven clinical utility for diseases such as cancer, the development and clinical implementation of biomarkers to personalize therapy in diabetes is lagging. Accumulating evidence indicates that extracellular vesicles (EVs) are key players in cell-to-cell communication and inter-organ crosstalk [6,7,8,9]. EVs carry unique signatures (e.g., proteins, lipids, nucleic acids) that are cell- and condition-specific [10,11,12]. Once released from cells, EVs can make their way into blood, urine, and other bodily fluids [11,13]. For these reasons, EVs are particularly attractive as biomarkers. Protein phosphorylation is a major regulatory mechanism in living cells and might provide important insight into function, but a number of challenges have limited the exploration of phosphoproteins as biomarkers, including the difficulty of reliably purifying and quantifying low-abundance phosphoproteins and interference from proteins and metabolites in the biofluids [14,15]. Recent advances in the development of EV-based technologies (i.e., using Extracellular Vesicle Total Recovery and Purification (EVtrap) beads followed by Polymer-based Metal Affinity Capture (PolyMAC), developed by Tymora Analytical Operations, Inc) for the characterization of the EV phosphoproteome may circumvent some of these limitations [16,17,18]. EVtrap is a recently developed, broad non-antibody-based affinity isolation method that produces quantitative EV yields that have proven advantageous for MS/MS proteomic and phosphoproteomic studies [16,17,18].

Recent evidence supports the role of EVs in the pathogenesis of T2DM [19,20,21,22,23] and underscores their biomarker and therapeutic potential [24,25]. However, little is known about the evolution of changes in EVs in the early stages of the human disease (i.e., PDM), and no characterization of the paired EV proteome and phosphoproteome across the diabetes spectrum exists. Therefore, the purpose of this study was to define the proteomic and phosphoproteomic landscape of circulating EVs in people with normal glucose tolerance, prediabetes, and type 2 diabetes. We additionally aimed to elicit mechanistic insight from the detected correlates among the circulating EV proteome, EV phosphoproteome, and relevant clinical measures assessing body composition, glucose control, and beta cell function in a well-phenotyped human cohort.

## 2. Results

### 2.1. Study Design and Clinical Characteristics of the Study Cohort

A balanced subset of patients from the ORIGINS study (ClinicalTrials.gov, ID: NCT02226640) was selected for this proteomics study (Table 1). Details from the parent study cohort have been previously described [26]. For this study, a total of 30 participants (*n* = 10 per group) were specifically selected as a subgroup that was not confounded by differences in sex, age, and obesity, which are known to affect metabolic function. Table 1 describes the summary of clinical characteristics of the study cohort.

### 2.2. EVs from Serum Are Highly Enriched with Exosomal Proteins and Phosphoproteins

**Figure 1:**
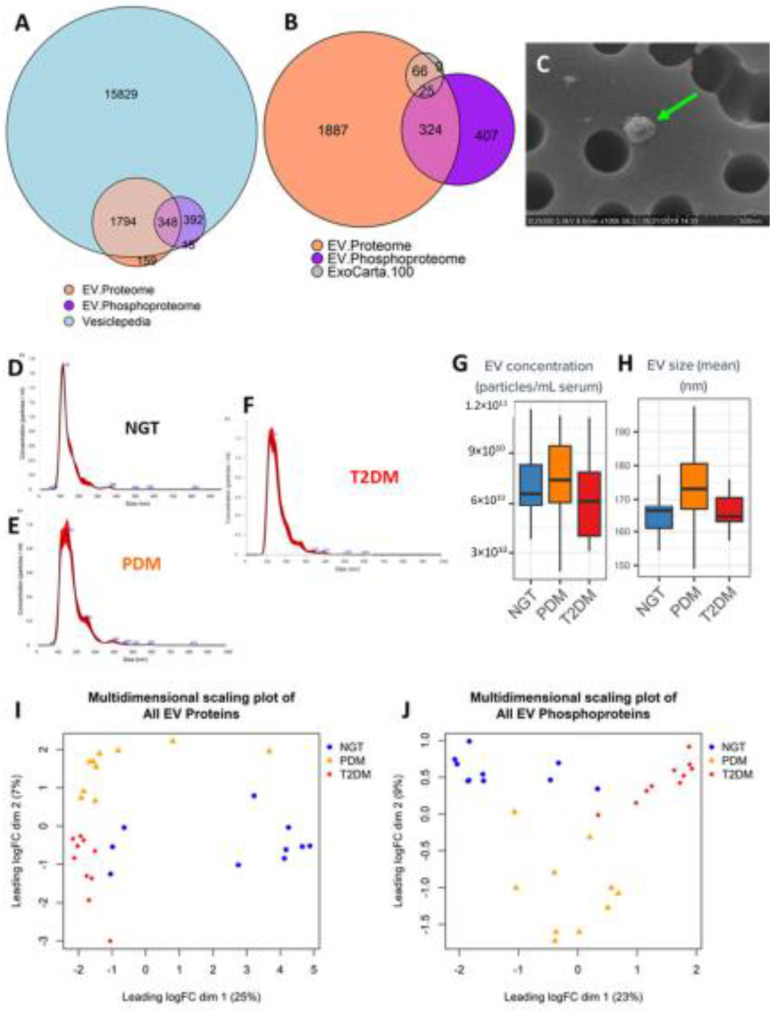
Characterization of EVs isolated from human serum from people with normal glucose tolerance (NGT), prediabetes (PDM), and type 2 diabetes mellitus (T2DM). (**A**,**B**) Euler diagram cross-referencing all reliably detected EV proteins and EV phosphoproteins to the Vesiclepedia database and to the list of best exosomal markers, as reported by Exocarta Top 100 database. Diagrams include the pool of all proteins and phosphoproteins detected in all samples. Total number of EV proteins and phosphoproteins in the Euler diagrams does not exactly add up to the total number of identified proteins because some Uniprot IDs (as reported by MS experiments) do not have associated gene names (as reported by Vesiclepedia and Exocarta). (**C**) Representative scanning electron microscopy of circulating EV, (**D**–**F**) Representative nanoparticle tracking analysis (NTA) traces of circulating EVs, depicting size distribution of particles in the 3 study groups. (**G**,**H**) Boxplots of total EV concentration and mean EV size, respectively. (**I**,**J**) Multidimensional scaling plots using all reliably detected EV proteins and phosphoproteins, respectively.

A total of 2372 unique EV proteins and 716 unique EV phosphoproteins were reliably identified (detected in at least any one of the samples with high confidence) from 1 mL of fasting serum that typically produces approximately 50–100 µg of total EV protein (EVs isolated using EVtrap technology). Most of these proteins and phosphoproteins have been reported to be present in EVs (by cross-referencing to the Vesiclepedia database, Figure 1A) and are specifically enriched in exosomal proteins (Figure 1B, 91% of the top 100 exosomal proteins reported as best markers of exosomes were readily identified). Scanning electron microscopy (SEM) of these preparations confirmed the presence of particles with morphology and dimensions consistent with those of exosomes (Figure 1C). Altogether, these data demonstrated that the EVtrap method significantly enriched the preparations with exosomes. We further characterized the distribution of particles by nanoparticle tracking analysis (NTA) and detected no significant differences in the total number of circulating EV-like nanoparticles among the three study groups (Figure 1D–H).

### 2.3. Differential Expression Analysis Provides Insight on Potential Tissue-Specific Mechanisms

**Figure 2:**
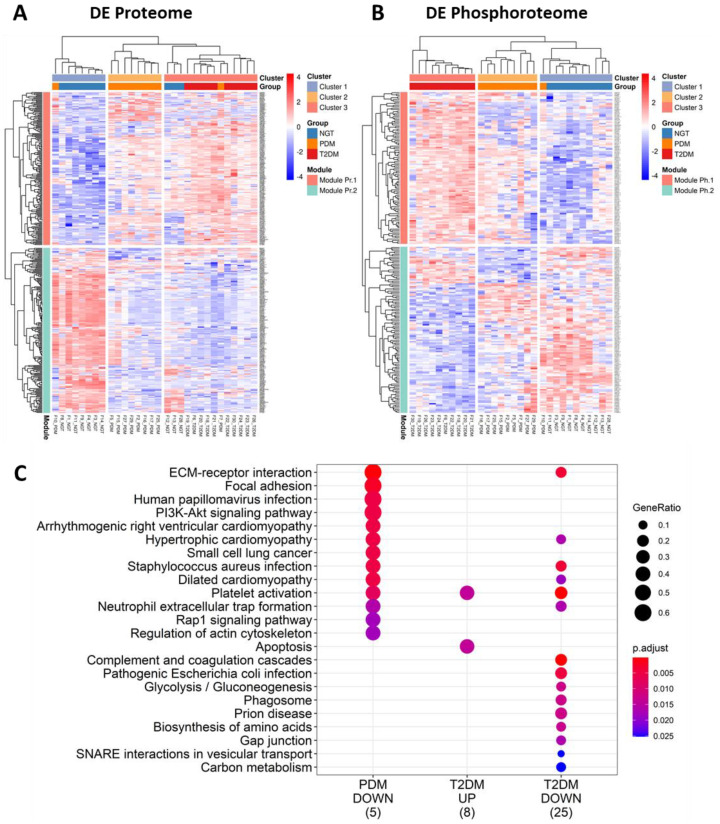
Differential expression analysis. (**A**,**B**) Heatmap of differentially expressed EV proteins (**A**) and EV phosphoproteins (**B**). Differentially expressed features required to have fold change greater than 2 (log2FC > 1 or log2FC < −1) and FDR < 0.05. Heatmaps were constructed using unsupervised clustering of the correlation distance between samples. Groups: NGT: normal glucose tolerance, PDM: prediabetes, T2DM: type 2 diabetes. Module.Pr.1: differentially expressed protein module 1, Module.Pr.2: differentially expressed protein module 2, Module.Ph.1: differentially expressed phosphoprotein module 1, Module.Ph.2: differentially expressed phosphoprotein module 2. Modules and clusters are generated by unsupervised clustering. (**C**) Enrichment of KEGG pathways among significantly different phosphoprotein/protein ratios. For the 245 features detected by both the proteomic and phosphoproteomic technologies, ratios were calculated, log-transformed, and compared using linear models that adjusted for sex, age, and BMI.

To gain insight into the biology underlying the development of diabetes and to identify potential circulating EV biomarkers of the disease, we implemented a multi-omic (proteomics and phosphoproteomics) approach to characterize the EV composition in serum (raw data tables are provided as Supplementary Data Tables SDT1–SDT3). Multidimensional scaling analysis of all the proteomic and phosphoproteomic data (Figure 1I,J) revealed that each study group presented relatively homogeneous profiles of EV proteins and phosphoproteins that were also distinguishable from the other groups. Consequently, we were able to identify 196 and 308 differentially expressed proteins, and 53 and 191 differentially expressed phosphoproteins in PDM and T2DM, respectively, as compared to NGT subjects (Supplementary Tables S1–S4). Supplementary Tables S5 and S6 report on the T2DM vs. PDM comparison for EV proteins and phosphoproteins, respectively. Using these circulating EV signatures and unsupervised clustering, we were able to correctly assign study participants to their respective groups with relatively high accuracy (Figure 2A,B).

**Figure 3:**
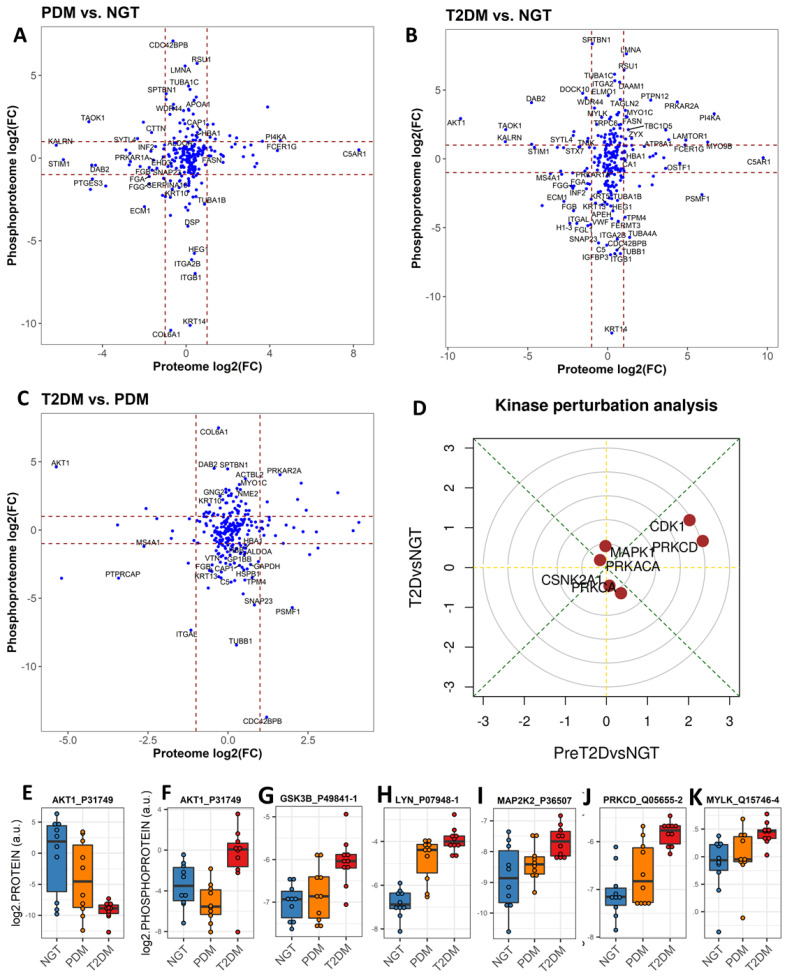
Global comparison of changes in the EV proteome and the EV phosphoproteome. (**A**) Correlation of changes in PDM, as compared to NGT. (**B**) Correlation of changes in T2DM, as compared to NGT. (**C**) Correlation of changes in T2DM, as compared to PDM. (**D**) Perturbation kinase analysis using the directPA package. (**E**–**K**) Interquartile range boxplots for differentially expressed total EV AKT1 protein and phosphorylated EV AKT1, GSK3B, LYN, MAP2K2, PRKCD, and MYLK kinases. Linear models adjusting for age, sex, and BMI were used to assess differential expression (fold change greater than 2, *p* < 0.05, and FDR < 0.05) using the limma R package.

Notably, the EV proteome and phosphoproteome displayed a relatively small number of simultaneously detected features (specifically, 245 proteins were also found as phosphorylated proteins in the circulating EVs) and a rather low correlation between the corresponding common features (Figure 3A–C). When normalized phosphoprotein/protein ratios were calculated for this subset of EV phosphoproteins, a striking pattern of mostly increased phosphorylation in PDM and mostly inhibition of phosphorylation in T2DM, as compared to the NGT group, was evident (Supplementary Tables S7–S9). The significantly different phosphoprotein/protein ratios, particularly those suggesting inhibition of phosphorylation (diminishing ratios when compared to the NGT group), were enriched for extracellular matrix-receptor interactions pathways, immune-related pathways involved in fighting infection, as well as PI3K-Akt signaling and Rap1 signaling pathways, among others (Figure 2A). The reduced phosphoprotein/protein ratios in T2DM additionally suggested impairment of metabolic pathways, as evidenced by the enrichment in pathways involved in glycolysis and gluconeogenesis and carbon metabolism (Figure 2C).

Of note, AKT1 was the phosphoprotein with the largest increase in phosphoprotein/protein ratio in T2DM (Supplementary Table S8). Intriguingly, the change in phosphorylated AKT1 in circulating EVs negatively correlated with the change in acute insulin response to glucose (AIRg), disposition index (DI), insulin secretion (HOMA-B), and glucose effectiveness (Sg) (r ≤ −0.39, *p* ≤ 0.05, Supplementary Table S10), while positively correlated with fasting plasma glucose (FPG), glucose AUC, and HbA1c (r ≥ 0.37, *p* ≤ 0.05, Supplementary Table S10). On the other hand, the change in total AKT1 protein significantly and expectedly negatively correlated with FPG, glucose AUC, and HbA1c (r ≤ −0.45, *p* < 0.013, Supplementary Table S11).

**Figure 4:**
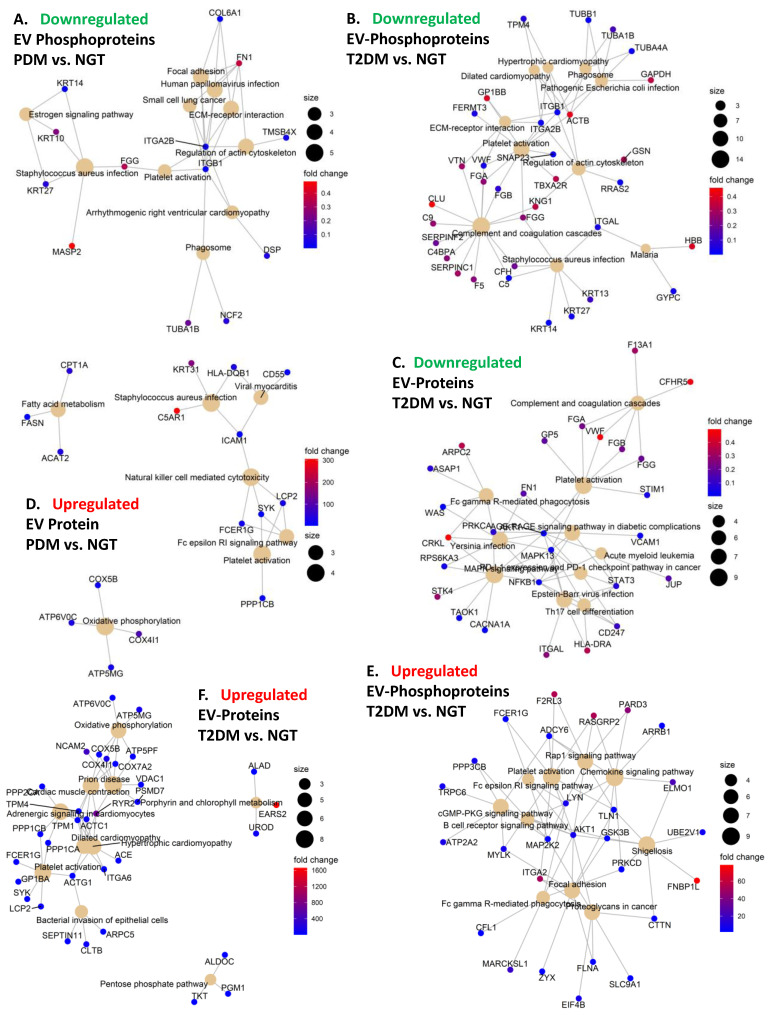
Pathway enrichment networks. (**A**,**B**) Network of enriched KEGG pathways in EV phosphoproteins that are downregulated in PDM compared to NGT (**A**) and in T2DM compared to NGT (**B**). (**C**) Network of enriched KEGG pathways in EV proteins that are upregulated in T2DM compared to NGT. (**D**) Network of enriched KEGG pathways in EV proteins that are upregulated in PDM compared to NGT. (**E**) Network of enriched KEGG pathways in EV phosphoproteins that are downregulated in T2DM compared to NGT. (**F**) Network of enriched KEGG pathways in EV proteins that are upregulated in T2DM compared to NGT.

In addition, signaling kinases including AKT1, GSK3B, LYN, MAP2K2, MYLK, and PRKCD were all among the significantly upregulated EV phosphoproteins (Figure 3F–K) that were central to a network enriched for immune-related pathways including chemokine signaling, Fc gamma R-mediated phagocytosis, and B cell receptor signaling, among others (Figure 4E). Similar to phosphorylated AKT1, the change in phosphorylated LYN and PRKCD kinases also significantly correlated with the change in FPG, glucose AUC, HbA1c, and the acute insulin response to glucose (AIRg) (absolute |r| ≥ 0.5, *p* < 0.12, Supplementary Table S10). On the other hand, the enrichment analysis of the differentially expressed EV proteins highlighted respective networks in both PDM and T2DM with significant enrichment in oxidative phosphorylation (OXPHOS) signaling among the upregulated proteins (Figure 4D,F). An enrichment in upregulated proteins involved in immune cell-mediating cytotoxicity was also detected in the PDM group (Figure 4D).

### 2.4. Phosphosite-Centric Analyses Identify Upstream Kinases Driving Phosphoproteomic Signatures in PDM and T2DM

To identify upstream active kinases responsible for the observed phosphoproteomic profiles in the circulating EVs, a kinase perturbation analysis [27] was conducted. This analysis predicts upstream-activated kinases based on the coordinated changes detected on their known substrates. As shown in the kinase perturbation plot in Figure 3D, we demonstrated that the activities of kinases CDK1 and PRKCD (PKCδ) were highly elevated in prediabetes (PDM vs. NGT comparison axis) and elevated, albeit not as dramatically, in T2DM (T2DM vs. NGT comparison axis). On the other hand, PRKCA and CSNK2A1 appear to be slightly inactivated in T2DM only.

**Figure 5:**
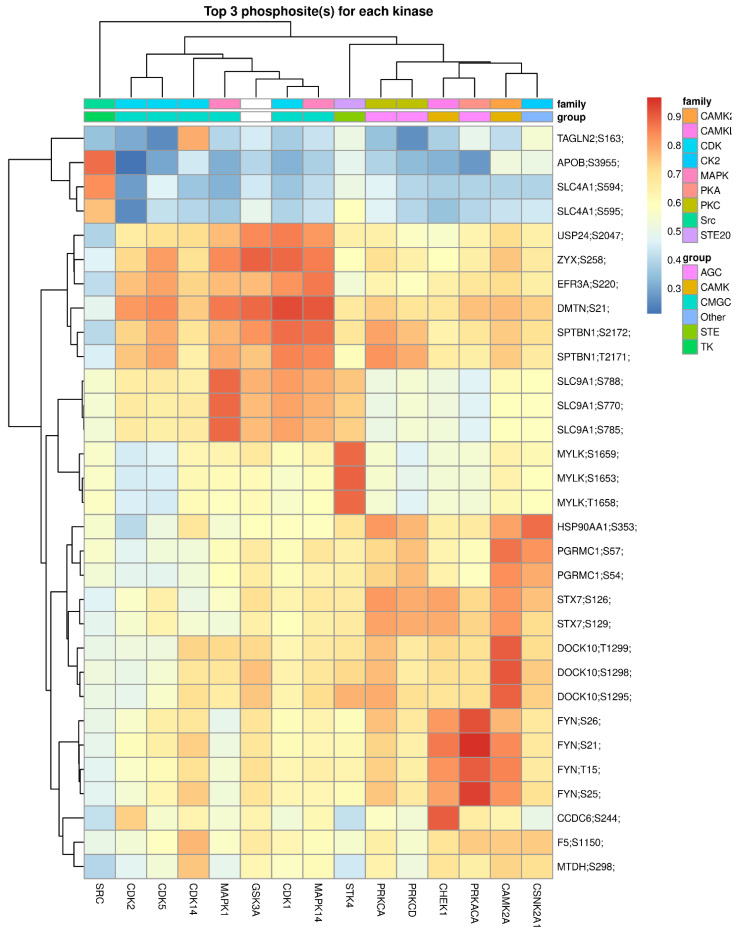
Global kinase-substrate relationships in the prediabetes and diabetes background. Only significantly upregulated phosphosites were included in the analysis. The heatmap shows the combined kinase-substrate scores for the top 3 phosphosites of all evaluated kinases. The higher the score, the better the fit of the phosphosite to a kinase motif and kinase-substrate phosphorylation profile.

To identify additional upstream kinases responsible for the phosphorylation of specific substrate sites and uncover potential novel kinase-substrate pairs (Figure 5, row dendrogram) and global relationships between kinases (Figure 5, column dendrogram), we used a multistep framework implemented in PhosR, a Bioconductor software package, to assesses both the likelihood of a kinase to recognize a specific motif and the dynamic phosphorylation profiles of the sites [28,29]. Several major kinase groups are associated with specific phosphorylation events in the prediabetic and diabetic backgrounds. Of note, a group of CMGC kinases (i.e., CDK1, GSK3B, MAPK1, MAPK14, CDK2, CDK5, and CDK14), as well as a mixed group of CAMK and AGC kinases (including two PKC isoforms, CHECK1, PRKACA, CAMK2A, and CSNK2A1), appear to have key non-redundant activities on specific substrates (Figure 5). Interestingly, upstream activation of STK4 (a major signaling kinase of the Hippo pathway [30]) appears to be the single kinase responsible for the increased phosphorylation of MYLK, one of the kinases that were central to the upregulated phosphoprotein network in T2DM (Figure 4E), as compared to the NGT group. Additional information on the differentially expressed phosphosites and pathways enrichment analysis results on those sets are provided in Supplementary Tables S12–S17.

### 2.5. Altered Expression of Platelet and Immune Activation and Coagulation Markers in Circulating EVs Is Common in PDM and T2DM

**Figure 6:**
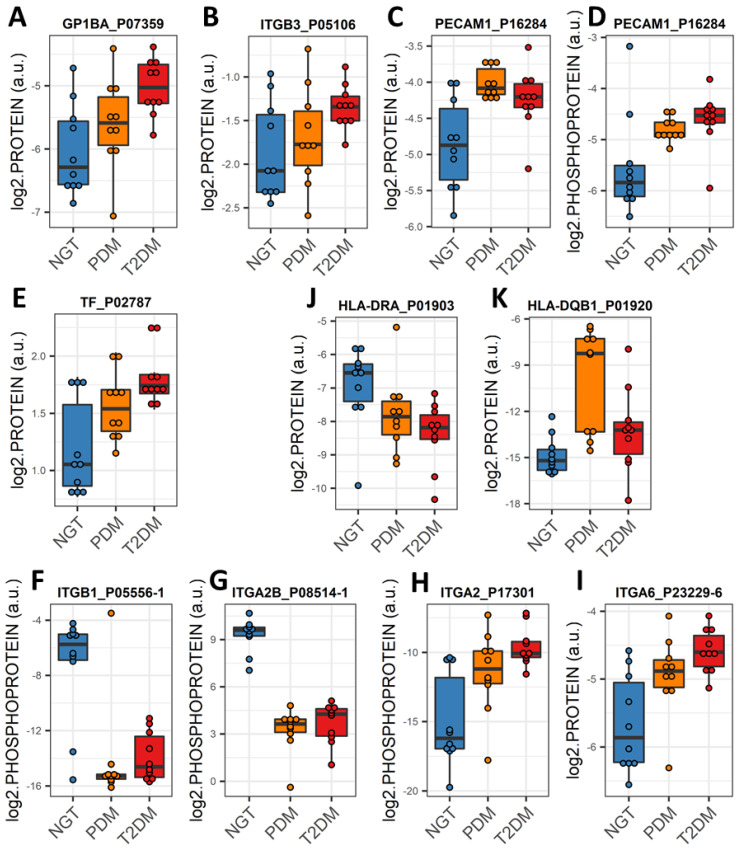
Expression profiles of select EV proteins and phosphoproteins. (**A**–**D**) Interquartile range (IQR) boxplots of differentially expressed EV proteins that are surface markers (**A**,**B**) and activation markers (**C**,**D**) of platelets. (**E**) IQR boxplot of differentially expressed tissue factor (TF), primary initiator of coagulation. (**F**–**I**) IQR boxplots of differentially expressed “switching” integrins. (**J**,**K**) IQR boxplots of differentially expressed major histocompatibility complex proteins in EVs. Linear models adjusting for age, sex, and BMI were used to assess differential expression (fold change greater than 2, *p* < 0.05, and FDR < 0.05) using the limma R package.

Proteins and phosphoproteins that play a role in chemokine signaling pathways in PDM and in platelet activation and coagulation in PDM and T2DM (Figure 4) were enriched among the differentially expressed EV proteins and phosphoproteins. Of note, significantly increased levels of the platelet surface markers GP1BA and integrin ITGB3 and the activation marker PCAM1 were common in EV preparations from both PDM and T2DM groups (Figure 6A–D). In addition, significant upregulation of tissue factor (TF) was also common in the circulating EVs from both groups (Figure 6E). TF in complex with coagulation factor F7a is the primary initiator of blood coagulation and has been reported to be released in EVs from platelets, monocytes, and pancreatic tumor cells, contributing to thrombus formation [31].

### 2.6. An “Integrin Switching” Signature in Circulating EVs Is Characteristic of PDM and T2DM

The networks of KEGG pathway-enriched downregulated EV phosphoproteins additionally highlighted a central role for integrins ITGB1 and ITGA2B in both the PDM and T2DM networks, as compared to the NGT group (Figure 4A,B and Figure 6F,G). Consequently, the modulation of interactions between the extracellular matrix (ECM) and cell receptors, the regulation of the actin cytoskeleton, and the regulation of phagosome functions were also among the key pathways enriched among the downregulated EV phosphoproteins (Figure 4A,B). On the other hand, phosphorylated ITGA2 and total ITGA6 protein were highly upregulated in the T2DM group (Figure 4E,F and Figure 6H,I), which suggests that T2DM might be associated with a switch of integrin surface molecules in EVs and, possibly, in the originating cells. Supporting our reasoning, HLA proteins, which are reported to modulate the expression of integrins [32], were also among the differentially expressed EV proteins highlighted by the functional enrichment networks (i.e., HLA-DRA and HLA-DQB1, Figure 4C,D and Figure 6J,K). Remarkably, phosphorylated integrins (i.e., ITGA2, ITGA2B, and ITGB1) correlated with important clinical measures of body composition (i.e., fat mass and waist circumference), glycemic control (i.e., FPG, glucose AUC, and HbA1c), glucose disposition (i.e., DI), insulin action (i.e., HOMA-IR), and beta cell function (i.e., fasting plasma insulin—FPI) (absolute |r| ≥ 0.4, *p* < 0.05, Supplementary Table S10).

### 2.7. Signatures of Liver Proteins and Phosphoproteins Are Downregulated in EVs as Early as the Prediabetes Stage

**Figure 7:**
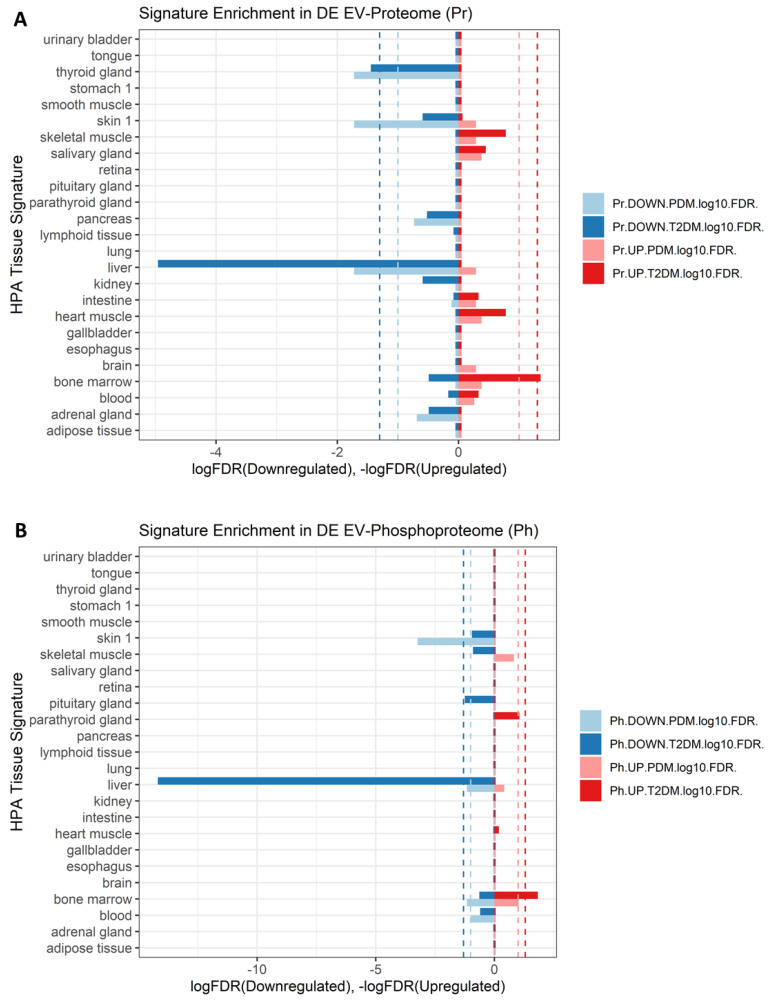
Enrichment for differentially expressed (DE) tissue-specific proteins (**A**) and phosphoproteins (**B**) in circulating EVs in people with prediabetes (PDM) and type 2 diabetes (T2DM), as compared to people with normal glucose tolerance (NGT). Pr: proteome; Ph: phosphoproteome; light blue color: downregulated features in PDM, dark blue color: downregulated features in T2DM; light red color: upregulated features in PDM, dark red color: upregulated features in T2DM. Darker discontinued lines at FDR = 0.05; lighter discontinued lines at FDR = 0.1.

**Figure 8:**
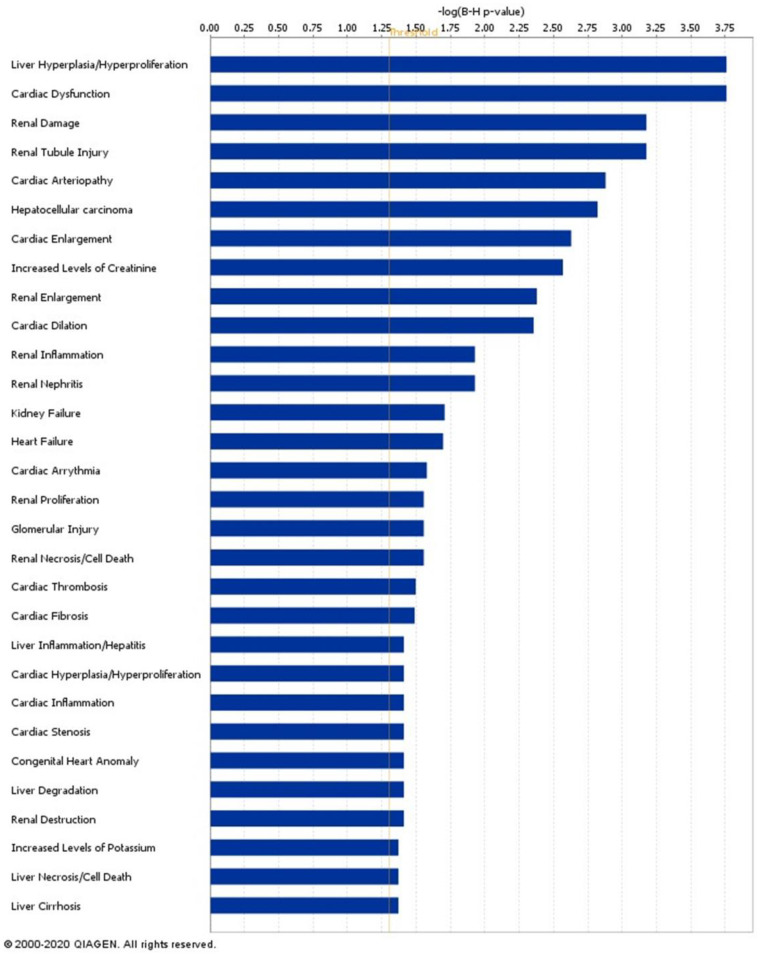
Downstream effects analysis (DEA) for differentially expressed proteins in the PDM group, as compared to the NGT group, using the Ingenuity Pathway Analysis (IPA) software. The analysis aims to identify biological functions that are expected to change in response to the protein expression patterns observed in the data. The algorithm is based on the calculation of an enrichment score and an activation Z-score [33].

To gain insight into which organs or cell types might be significantly contributing to the differences in EV protein and phosphoprotein cargo in PDM and T2DM, we conducted enrichment analyses for cell type-specific signatures extracted from the Human Protein Atlas (HPA). As shown in Figure 7, we detected a highly enriched (FDR <<< 0.05) signature of liver-specific markers among the downregulated EV proteins and EV phosphoproteins in both the PDM and T2DM groups, as compared to NGT. Moreover, by conducting downstream effects analysis (DEA) using Ingenuity Pathway Analysis (IPA) software, we observed that the differentially expressed EV proteome in PDM appears to code for suppression of liver cell death and hyperproliferation functions with concomitant activation of liver inflammatory processes (Figure 8). On the other hand, the same EV proteome seems to code for the downstream activation of renal damage and necrotic cell death processes in connection with renal nephritis and kidney failure pathways (Figure 8).

## 3. Discussion

As a complex disease, diabetes development and progression involve many alterations in a variety of signaling pathways and biological processes that are often difficult to summarize in a single-theme narrative, for example, those describing multiomic studies such as this one. However, the multiomic approach can provide a large body of mechanistic insight that confirms previous and contribute new knowledge to the field and warrant the development of important validation studies.

Understanding of the role of exosomes and EVs in type 2 diabetes has evolved in the last decade. However, most EV profiling studies to date have focused on the miRNA cargo. To our knowledge, no study has addressed the simultaneous quantification of circulating EV proteins and phosphoproteins. EV phosphoproteomics has been conducted in the cancer field using cultured cells, with a small number of studies characterizing the phosphoproteome of EVs from urine or plasma/serum samples from healthy people or people with other conditions [16,18,34,35,36,37]. Thus, our work makes an important contribution by defining the proteomic and phosphoproteomic landscape of circulating EVs in prediabetes and diabetes.

In addition to identifying EV proteomic and phosphoproteomic signatures across the spectrum of diabetes, our data suggest potential EV-mediated mechanisms that might underlie the development of prediabetes and diabetes and its complications. These associations are, of course, limited by the cross-sectional nature of this study. As shown in Figure 1, unsupervised clustering of select EV protein and phosphoprotein signatures could separate the study participant samples based on their disease stage with relatively large accuracy. The features detected by both our proteomic and phosphoproteomic experiments and with significantly different phosphoprotein/protein ratios, particularly those suggesting inhibition of phosphorylation, were enriched for extracellular matrix-receptor interactions pathways, immune-related pathways involved in inflammation and fighting infection, and in PI3K-Akt and Rap1 signaling as well as relevant metabolic processes, among others. Inactivation of both the PI3K-Akt and the Epac2/Rap1 signaling pathways appears highly relevant to the development of diabetes because the pathways have been reported to be essential for mediating survival, proliferation, glucose homeostasis, and lipid metabolism, among others (PI3K-Akt) [38] and for regulation of insulin granule dynamics by cAMP and insulin secretion (Epac2-Rap1) [39,40,41]. Moderately inducing the PI3K-Akt signaling pathway in pancreatic β cells has, in fact, been suggested as a therapeutic strategy to preserve β cell mass during the development of type 1 and type 2 diabetes [42].

Intriguingly, we detected a dramatic change in phosphorylated AKT1 (which significantly increases in circulating T2DM EVs while the total protein level diminishes), and these changes are associated with the change in relevant clinical measures of glycemic control, insulin secretion, and insulin action. We reason that AKT1 phosphorylation increases in T2DM are likely to compensate for the dramatic reduction in total AKT1 protein and activity. However, a thorough analysis of phosphorylation sites and their effect on kinase activity is warranted, as not all phosphorylation sites are associated with a similar protein activation. This finding is significant because phospho-AKT is a key signaling molecule downstream of the insulin receptor, and “the substrates of AKT are intimately linked to the various physiological functions of insulin and are often specific to a particular cell type” [43]. Interestingly, impaired translocation and activation of mitochondrial AKT1 reduced the activity of mitochondrial OXPHOS Complex V in diabetic myocardium [44,45], and oxidative phosphorylation was found to be overrepresented among the differentially expressed EV proteins and phosphoproteins in our study (Figure 4D,F). Notably, oxidative stress has been demonstrated to be a causal factor in the impairment of adipose tissue metabolism and insulin resistance [46,47]. Proteins involved in oxidative phosphorylation are known to be reduced in various tissues in diabetes [48,49,50,51], but, surprisingly, we detected them upregulated in the circulating EVs. We reason, then, that an abnormal removal of OXPHOS-related proteins and phosphoproteins via EV secretion (e.g., in adipocytes from people with obesity) might underlie the development of diabetes. It is tempting to speculate that abnormal EV-mediated disposal function may be diverting phospho-AKT1 from translocating to the mitochondria.

Other significantly upregulated phosphorylated kinases in the circulating EVs in the same pathway-enriched network as AKT1 included GSK3B, LYN, MAP2K2, MYLK, and PRKCD. Two of these kinases (namely, PRKCD and GSK3B) were also identified by our phosphosite-centric analyses as diabetes-related upstream kinases contributing to the observed global patterns of substrate-kinase relationships (see kinase perturbation plot in Figure 3 and kinase heatmap on Figure 5). These kinases coordinate signaling of multiple pathways involved in immune cell activation, among others, which were overrepresented among differentially expressed EV proteins and phosphoprotein networks. Supporting our findings, these pathways have been shown to play important roles in obesity-associated insulin resistance, among other diabetes-associated phenomena [52,53,54].

The kinase perturbation analysis based on composite scores of kinase-substrate profiles provided important evidence to predict which kinases appear to produce the EV phosphoproteomic signatures observed in PDM and T2DM. These results contribute to our understanding of pathophysiological processes taking place during diabetes development in humans. For example, we speculate that the highly elevated upstream activities of CDK1 and PKCδ (represented by its catalytic subunit PRKCD) in the prediabetes stage may contribute to the development of diabetes. Supporting our reasoning, it was recently discovered that, independent of its cell cycle functions, CDK1 acts as a regulator of mitochondrial complex I and enhances β cell glucose sensing in the pancreas [55]. However, persistent activation of CDK1 during obesity causes both beneficial and pathological consequences for the pancreatic beta cell in mice, including reduction in their insulin secretory capacity that is recovered by pharmacologic inhibition with RO-3306 [55]. Similarly, family member CKD2 also contributes to switching off the β cell secretory capacity [56]. On the other hand, PKCδ has been reported to promote insulin secretion from the pancreatic β cell by increasing the number of insulin granules in the ready-releasable pool [57] and to be elevated in the liver of mice and humans with obesity, where it plays an important role in the development of hepatic insulin resistance and hepatosteatosis [58,59]. Now, our phosphoproteomic study profiling human circulating EVs confirms the activation of CDK1 and PKCδ in humans with prediabetes and diabetes and suggests clinical utility for phosphoproteomics of circulating EVs. Future prospective studies addressing the diagnostic and prognostic capacity of these two kinases are warranted.

The kinase-substrate heatmap also uncovered that STK4 (a major signaling kinase of the Hippo pathway [30]) appears to be the kinase responsible for the increased phosphorylation of MYLK, a myosin light chain kinase that is involved in Ca^2+^ signaling, myofibroblast contraction, microvascular endothelial barrier dysfunction, gastric motility, and insulin secretion [57,60,61,62]. Supporting our finding, altered contractility of the gastric smooth muscles has been found impaired in people with obesity and diabetes [60]. Remarkably, three of the kinases identified by our study (i.e., PKA, PKC, and MYLK) interact in a synergistic way to promote insulin secretion from the pancreatic β cell by increasing the number of insulin granules in the ready-releasable pool [57].

The differentially expressed proteins and phosphoproteins were also significantly enriched in platelet activation and coagulation effectors. This is consistent with the known crosstalk between coagulation and inflammation in diabetes, which is a key determinant in the development of complications such as cardiovascular disease, diabetic nephropathy, and retinopathy, among others [63,64,65]. Moreover, we also detected a significant reduction in phosphorylated integrins ITGB1 and ITGA2B in the circulating EVs, while phosphorylated ITGA2 and total ITGA6 protein were significantly upregulated. Of note, HLA proteins have been reported to modulate the expression of integrins [32], and we found HLA-DRA and HLA-DQB1 coordinately and significantly changing and highlighted in the functional enrichment networks. We speculate that these changes may indicate a switch of integrin surface markers in cells and their released EVs, which may consequently change their interaction patterns with the surrounding ECM and tropism for the circulating EVs, respectively [66,67]. The fact that these EV proteins and phosphoproteins were identified as central hubs in their respective pathway enrichment networks and that their changes significantly correlated with changes in relevant clinical variables, including measures of body composition, glycemic control, insulin action, and beta cell function, suggests that their presence and changes in abundance in the circulating EVs is important in the development of T2DM. For example, the positive correlation between upregulated phosphorylated integrin ITGA2 and glucose AUC, FPG, and HbA1c may suggest that the progressively increased phosphorylation of ITGA2 plays a role in (or is a consequence of) the deterioration of glycemic control as subjects progress from NGT to PDM to T2DM. On the other hand, the negative correlations between downregulated integrins ITGB1 and ITGA2B and the same glycemic control variables and others related to body composition and insulin resistance suggest a similar role in the inhibition of phosphorylation in these kinases. We speculate that the apparent integrin switch is potentially triggered by the phosphorylation events occurring on those three integrins. In support of our reasoning, the crosstalk between integrins conducing to the activation or inactivation of its function is mediated, at least in part, by phosphorylation of the β-chains, and phosphorylation switches may induce the crosstalk between integrins and other receptors [68]. However, our evidence is only correlative; therefore, we cannot determine causality. Remarkably, ECM-integrin signaling (e.g., due to increased density of the collagen-binding integrin α2β1 dimer, a key collagen-binding receptor in the plasma membrane of platelets and muscle cells, among others) has been associated with muscle insulin resistance [69,70], inflammation [71], angiogenesis [72], and cardiovascular risk [73], among others. Our study did not aim to quantify the levels of integrin heterodimers, but the fact that the phosphorylated α2 (ITGA2) subunit was elevated in the circulating EVs of both PDM and T2DM subjects may indicate elevated density and/or activity of its heterodimers in specific tissues and secreted EVs. Consistent with its role in platelet function, platelet activation was among the pathways most commonly and significantly enriched among the differentially expressed proteins and phosphoproteins detected in this study in both PDM and T2DM subjects. On the other hand, altered density or activity of integrin molecules such as integrin β1 (ITGB1) have been implicated in a variety of diabetic complications, particularly diabetic nephropathy [74,75,76]. ITGB1 has been found to be particularly important in podocytes, where it mediates signaling in the axis IGFBP1/ITGB1/FAK and contributes to essential podocyte functions by promoting cell adhesion, motility, and survival [77]. The activity of this axis was found to be controlled by FOXO1, which was in turn inhibited by activated insulin-PI3K-AKT signaling [77]. Interestingly, we found phosphorylated AKT upregulated in the circulating EVs from the T2DM group. Moreover, the activity of the axis was demonstrated to be reduced in glomeruli from humans with early type 2 diabetic kidney disease. Supporting the key role of ITGB1 in the kidneys, the β1-integrin-knockout mice develop severe proteinuric kidney disease from birth [78], and patients with Abatacept-stabilized β1-integrin activation are protected from B7-1-positive proteinuric kidney disease [79]. Remarkably, Ingenuity DEA analysis suggested that the differentially expressed EV proteome identified in people with PDM in our study is associated with the activation of pathways involved in renal damage and necrotic cell death, as well as in renal nephritis and kidney failure. Altogether, our data now suggest that profiling the integrin composition of circulating EVs could aid in the early detection of diabetic complications.

Another novel and important finding from our study is the highly significant downregulation of circulating EV proteins and phosphoproteins from the liver in both the PDM and T2DM groups. This defect may be caused by (1) decreased expression of proteins and phosphoproteins in liver cells, hence reduced levels in the liver-derived EVs, (2) by decreased packaging of proteins and phosphoproteins into EVs, (3) by reduced secretion of EVs with neither a defect in EV packaging nor in cytoplasmic levels of the specific proteins and phosphoproteins, or (4) by some combination of the previous three defects. With the data at hand, we are unable to dissect the specific cause for the alterations in EV cargo composition in the liver or any other tissue. However, the fact that additional analyses (i.e., DEA) suggested that the differentially expressed EV proteins are involved in the suppression of liver cell death and hyperproliferation functions with concomitant activation of liver inflammatory processes in PDM supports an important early role for the liver (potentially mediated by alterations in EV cargo and inter-organ cross-communication) during diabetes development. Indeed, the important role of the liver in diabetes is well established and elegantly incorporated in the twin cycle hypothesis [80,81]. Our study now suggests that alterations in the cargo and/or in the number of liver-derived circulating EVs represent early pathophysiological changes that could serve as biomarkers of disease development. These EV protein dynamics, identifiable as early as in prediabetes, appear to represent early events contributing to the known increased risk of developing steatosis and renal fibrosis in subjects with type 2 diabetes [82,83,84]. Notably, important links between liver, kidney, and heart pathologies have been reported, and the pathogenic crosstalk between the liver and the inflamed adipose tissue is widely accepted [85,86]. Our data add support for the role of circulating EVs in this pathogenic crosstalk, pointing at the liver and kidneys as early partners in crime.

Contrasting with our finding that no significant changes occur in the total concentration of circulating EVs in diabetes, other authors have reported increased numbers of circulating particles [21]. However, their EV isolation methods (i.e., polymer precipitation and ultracentrifugation) are less specific than the EVTRAP method employed in this study. Polymer precipitation and ultracentrifugation are known to isolate other types of contaminating particles that could mistakenly be counted as EVs. On the other hand, using flow cytometry quantification of blood cell-specific markers, a significant increase in erythrocyte-derived EVs was associated with diabetes [21]. This latter finding is in agreement with the significant enrichment that we observe in bone marrow cell-specific markers among the upregulated EV proteins and phosphoproteins in people with T2DM (Figure 6).

Our study has limitations, including the small sample size, the cross-sectional nature, the lack of functional measures or imaging of the liver and kidneys, and the fact that we cannot experimentally separate out tissue-specific EVs. Despite these limitations, the study has several strengths, including the use of a well-phenotyped human cohort, careful control for confounding factors, balanced sex, and the use of state-of-the-art methods for broad non-antibody-based specific EV isolation, proteomics, phosphoproteomics, and EV bioinformatics. As a complex disease, diabetes is likely the consequence of many alterations in a variety of signaling pathways and biological processes. This study demonstrated that an impressive number of proteome and phosphoproteome alterations are detectable as early as the prediabetes state, with consistent patterns of systemic kinase activity. Importantly, we uncovered sets of potentially activated kinases that appear to drive specific protein phosphorylation signatures during diabetes development and/or progression. These kinases represent potential novel therapeutic targets against diabetes. Furthermore, phosphorylated kinases including PKCδ, AKT1, GSK3B, LYN, MAP2K2, and MYLK are found circulating in EVs, hence with potential for long reach systemic action via inter-organ communication. The role of the liver, with an apparently impaired EV protein and phosphoprotein output in both prediabetes and diabetes, is also evidenced by our study. Some of the markers identified by this work could be studied as early predictors of diabetes development and/or progression and potentially indicate the need for aggressive preventive strategies.

## 4. Materials and Methods

### 4.1. Samples

All procedures were approved by the AdventHealth Translational Research Institute Institutional Review Board. Informed consent was obtained from all volunteers before the initiation of the study. Archived serum samples from 30 human subjects (*n* = 10 per group, ORIGINS study, ClinicalTrials.gov NCT02226640). The groups were selected, according to ADA guidelines [87], to have either normal glucose tolerance (NGT), prediabetes (PDM), or T2DM. Subjects with type 1 diabetes or other types of diabetes were not included in the ORIGINS study. The inclusion and exclusion criteria of the subjects were described previously (ClinicalTrials.gov, ID: NCT02226640). Participants were specifically selected as a subgroup that was relatively well balanced for sex, age, and obesity. This selection was partially automated using a custom script based on the *MatchIt* package [88] in the R programing environment through a series of recursive pairwise propensity score matching cycles (between two of the study groups at a time) until all pairwise comparisons were exhausted and the desired sample size was achieved).

### 4.2. Clinical and Metabolic Measurements

Anthropometric measures were performed according to standardized protocols. Body composition was measured using a GE Lunar iDEXA whole-body scanner (GE, Madison, WI, USA). Fasting blood samples were obtained, and subjects underwent a 2 h 75 g oral glucose tolerance test (OGTT). On a different visit, an insulin-modified frequently sampled intravenous glucose tolerance test (FSIVGTT) [89] was performed. Plasma glucose concentrations were measured using the glucose oxidase method with a YSI 2300 STAT Plus Analyzer (YSI Life Sciences, Yellow Springs, OH, USA). Plasma insulin and C-peptide concentrations were determined using the MSD human insulin assay kit and C-peptide kit, respectively (MSD, Rockville, MD, USA). HbA1c levels were measured using a Cobas Integra 800 Analyzer (Roche, Basel, Switzerland). The β cell function was assessed by calculating HOMA-B, the insulinogenic index [ΔIns_0–30′_/ΔGluc_0–30′_], and the insulin and C-peptide areas under the curve (AUC) in response to OGTT. Insulin activity was assessed by calculating HOMA-IR as described elsewhere. Data from the FSIVGTT were used to calculate insulin sensitivity (Si) and acute insulin response to glucose (AIRg) using the Minimal Model method of Bergman [90].

### 4.3. EV Isolation

EV purification was conducted using EVtrap (Tymora Analytical, Lafayette, IN, USA), a non-antibody-based affinity technology developed by Tymora to specifically and quantitatively isolate EVs. In short, frozen serum samples were thawed, and any large debris was removed by centrifugation at 2500× *g* for 10 min. The pre-cleared plasma samples were then diluted 20-fold in PBS and incubated with EVtrap beads for 30 min [17]. After supernatant removal using a magnetic separator rack, the beads were washed with PBS, and the EVs were eluted by a 10 min incubation with 200 mM triethylamine (TEA, Millipore-Sigma), and the resulting EV samples were fully dried in a vacuum centrifuge.

### 4.4. Mass Spectrometry (LC-MS/MS)-Based Methods Developed by Tymora Were Used to Detect the Global EV Proteome and Phosphoproteome

The isolated and dried EV samples were processed as described previously [16]. Briefly, EV samples were lysed to extract proteins using the phase-transfer surfactant (PTS) aided procedure [91], and the proteins were digested with Lys-C (Wako) at 1:100 (*wt/wt*) enzyme-to-protein ratio for 3 h at 37 °C. Trypsin was added to a final 1:50 (*wt/wt*) enzyme-to-protein ratio for overnight digestion at 37 °C. After surfactant removal, the resulting peptides were desalted using Top-Tip C18 tips (Glygen, Columbia, MD, USA) according to the manufacturer’s instructions. Each sample was split into 99% and 1% aliquots for phosphoproteomic and proteomic experiments, respectively. The samples were dried completely in a vacuum centrifuge and stored at −80 °C. For phosphoproteome analysis, the 99% portion of each sample was subjected to phosphopeptide enrichment using PolyMAC Phosphopeptide Enrichment kit (Tymora Analytical, West Lafayette, IN, USA) according to the manufacturer’s instructions, and the eluted phosphopeptides dried completely in a vacuum centrifuge. For phosphoproteomics analysis, the whole enriched sample was used, while for proteomics, only 50% of the sample was loaded onto the LC-MS.

Each dried peptide or phosphopeptide sample was dissolved at 0.1 μg/μL in 0.05% trifluoroacetic acid with 3% (*vol/vol*) acetonitrile. A total of 10 μL of each sample was injected into an Ultimate 3000 nano UHPLC system (Thermo Fisher Scientific, Waltham, MA, USA). Peptides were captured on a 2 cm Acclaim PepMap trap column and separated on a heated 50 cm column packed with ReproSil Saphir 1.9 μm C18 beads (Dr. Maisch GmbH, Ammerbuch, Germany). The mobile phase buffer consisted of 0.1% formic acid in ultrapure water (buffer A) with an eluting buffer of 0.1% formic acid in 80% (*vol/vol*) acetonitrile (buffer B) run with a linear 60 min gradient of 6–30% buffer B at a flow rate of 300 nL/min. The UHPLC was coupled online with a Q-Exactive HF-X mass spectrometer (Thermo Fisher Scientific, Waltham, MA, USA). The mass spectrometer was operated in the data-dependent mode, in which a full-scan MS (from *m*/*z* 375 to 1500 with a resolution of 60,000) was followed by MS/MS of the 15 most intense ions (30,000 resolution; normalized collision energy—28%; automatic gain control target (AGC)—2E4, maximum injection time—200 ms; 60 sec exclusion).

### 4.5. Bioinformatic Analysis of MS/MS Data

The raw files were searched directly against the human Uniprot database with no redundant entries, using Byonic (Protein Metrics, Cupertino, CA, USA) and Sequest search engines loaded into Proteome Discoverer 2.3 software (Thermo Fisher Scientific, Waltham, MA, USA). MS1 precursor mass tolerance was set at 10 ppm, and MS2 tolerance was set at 20 ppm. Search criteria included a static carbamidomethylation of cysteines (+57.0214 Da), variable modifications of oxidation (+15.9949 Da) on methionine residues, acetylation (+42.011 Da) at the *N* terminus of proteins, and phosphorylation of S, T, and Y residues (+79.996 Da) for the phosphoproteomics data. The search was performed with full trypsin/P digestion and allowed a maximum of two missed cleavages on the peptides analyzed from the sequence database. The false discovery rates of proteins and peptides were set at 0.01. All protein and peptide identifications were grouped, and any redundant entries were removed. Only unique peptides and unique master proteins were reported.

All data were quantified using the label-free quantitation node of Precursor Ions Quantifier through the Proteome Discoverer v2.3 (Thermo Fisher Scientific, Waltham, MA, USA). For the quantification of proteomic or phosphoproteomic data, the intensities of peptides/phosphopeptides were extracted with initial precursor mass tolerance set at 10 ppm, minimum number of isotope peaks as 2, maximum ΔRT of isotope pattern multiplets—0.2 min, PSM confidence FDR of 0.01, with hypothesis test of ANOVA, maximum RT shift of 5 min, pairwise ratio-based ratio calculation, and 100 as the maximum allowed fold change. The abundance levels of all peptides and proteins were normalized using the total peptide amount normalization node in the Proteome Discoverer. For calculations of fold change between the groups of proteins, total protein abundance values were added together, and the ratios of these sums were used to compare proteins within different samples.

### 4.6. Nanoparticle Tracking Analysis (NTA)

The size distribution and concentration of particles in EV preparations were analyzed using dynamic light-scattering technology with a NanoSight NS300 instrument and NTA-3.4 software (Malvern Panalytical, Malvern, UK). The instrument was equipped with a 488 nm blue laser module, flow-cell top plate, integrated temperature control, and a single-syringe pump module. Samples were diluted using cell culture-grade water (Corning cat# 25-005-CI, Mediatech Inc., Manassas, VA, USA) to produce an optimal particle concentration for final measurement in the range of 10^7^ to 10^9^ particles/mL. Final quantification included 5 standard measurements of 1 min of duration each, taken at a controlled temperature of 25 °C and under constant recommended automatic flow. The camera level for video capture was set to 12 and the detection threshold to 5 for all sample measurements.

### 4.7. Scanning Electron Microscopy (SEM) 

Representative SEM images of EV samples were obtained at the Interdisciplinary Center for Biotechnology Research Electron Microscopy Core Laboratory at the University of Florida. In short, EV preps were fixed in Trump’s fixative for 30 min at room temperature, then spread onto Isopore 0.2 µm GTTP filters (Merck Millipore, Tullagreen, Ireland) that had been pre-treated with 0.01% poly-L-lysine solution. The filters were washed 3 times with 1x PBS and 3 times with filter-sterilized deionized water. The fixed EVs were microwave-stabilized in a Pelco Biowave (Ted Pella, Redding, CA, USA), then dehydrated through an ethanol series at 10, 25, 50, 75, 90, and 100%, followed by critical-point drying (CPD) using a Tousimis CPD system (Tousimis, Rockville, MD, USA). Samples were sputter-coated with gold-palladium and imaged with a Hitachi SU5000 Schottky Field-Emission SEM (Hitachi High-Technologies, Schaumburg, IL, USA).

### 4.8. Phosphosite-Centric Analyses

Phosphosite level analysis was conducted using PhosR, a Bioconductor package [29] in the R 4.1.2 environment. Code for processing and downstream analyses of the phosphoproteomic data was adapted from reference [28] and the PhosR package vignette. PhosR uses a multistep framework to assess both the likelihood of a kinase recognizing a specific motif and the dynamic phosphorylation profiles of sites [28]. Upstream kinases responsible for the phosphorylation of specific detected phosphosites are identified, and potential novel kinase-substrate pairs and global relationships between kinases are uncovered. In summary, phosphosite filtering (forced to be present in at least 50% of the samples from one study group) and imputation (site and condition-specific imputation with scImpute using 50% as quantification rate threshold per condition and tail-base imputation with tImpute functions) was performed. We then implemented quantile normalization of the imputed data using the limma Bioconductor package and conducted differential phosphosite expression analysis using linear models and moderated empirical Bayes statistics implemented in limma [92,93]. Kinase perturbation analysis was then conducted using the perturbPlot2d function from the directPA R package [27]. DirectPA tests the combined effects of different treatments (conditions in our case) by rotating polar coordinates in two-dimensional space when the experiment contains two perturbations (PDM and T2DM groups) and corresponding controls (NGT group). For kinase-substrate prediction analysis conducted using the PhosR package, the normalized expression data were standardized, filtered for upregulated phosphosites, and used for the calculation of combined kinase-substrate scores based on knowledge of kinase recognition motifs, phosphoproteomics dynamics, and prediction of novel kinase-substrate relationships using an adaptive sampling-based positive-unlabeled learning method [28]. The PhosphoSitePlus annotation database was used as the source of kinase-substrate annotations [94].

### 4.9. Detection of Tissue-Specific Signatures and KEGG Pathway Enrichment Analysis

The lists (signatures) of tissue-specific proteins were downloaded from the Human Protein Atlas [95] (https://www.proteinatlas.org/humanproteome/tissue/tissue+specific, accessed on 12 June 2021). Enrichment for the tissue-specific signatures among the lists of differentially expressed EV proteins and phosphoproteins was assessed via the implementation of the hypergeometric test using the phyper function from the stats R package. The complete list of proteins reported by Vesiclepedia [96] plus the additional novel EV proteins detected by our proteomics experiments were used as background for the hypergeometric tests. Enrichment of KEGG pathway annotations among the sets of differentially expressed proteins and phosphoproteins was assessed using clusterProfiler [97] with a *p* < 0.05 and adjusted *p* < 0.1 as thresholds for statistical significance.

### 4.10. Downstream Effect Analysis (DEA)

Using the Ingenuity^®^ Pathway Analysis (IPA) software (QIAGEN, Redwood City, CA, USA), we conducted DEA [33]. In summary, this analysis aims to identify biological functions that are expected to change in response to the gene or protein expression patterns observed in the data. The analysis is based on the calculation of “two scores that address two independent aspects of the inference problem: an ‘enrichment’ score (Fisher’s exact test (FET) *p*-value) that measures overlap of observed and predicted regulated gene sets, and a Z-score assessing the match of observed and predicted up/down regulation patterns.” [33]. The activation Z-score is based on the user’s profiling data and prior knowledge of expected causal effects between genes and biological functions stored in the Ingenuity^®^ Knowledge Base (QIAGEN, Redwood City, CA, USA).

### 4.11. Statistical Analysis

Data normality was tested using the Shapiro–Wilk test, and nonnormal data were log-transformed to approximate normality. Differences in baseline clinical characteristics were assessed using the Welch two-sample t-test (for continuous variables) or the Fisher exact test (for categorical variables). For assessment of differential expression in EV-shuttled proteins and phosphoproteins, linear models using the *limma* package [92] were implemented. Our models included age, sex, and BMI as established covariates. Partial correlations were also calculated in the R environment, adjusting for the same covariates. Post-hoc analysis was performed using the *phia* package. Calculated effects with fold change greater than 2 (log2FC > 1 or log2FC < −1 and adjusted *p*-value < 0.05) and correlations with two-tailed *p* < 0.05 were considered significant. False discovery rates (FDR) correcting for multiple testing were calculated using the Benjamini–Hochberg correction as implemented for the *p.adjust* function in the *stats* package.

## 5. Conclusions

This work makes an important contribution toward defining the proteomic and phosphoproteomic landscape of circulating EVs across the diabetes disease spectrum. Among key findings, our data indicate that reduced levels of AKT1 protein but with increased phosphorylation status in circulating EVs, likely preceded by activation of CDK1 and PKCδ kinases (among others) since the prediabetes stage, may underlie the development of pathogenic events conducing to diabetes. Among other characteristic common changes in the prediabetic and diabetic EVs, “integrin switching” appear to be a central feature of functional enrichment networks with a potential impact on disease development and the known increased risk for complications. In addition, a highly significant signature of downregulated liver-specific EV proteins is demonstrated in both the EV proteome and phosphoproteome as early as prediabetes. This suggests a reduced EV output from the liver, among other possible causes, due to an impaired endocytic secretory pathway in the early stages of disease development. Suppressed liver cell death functions contrasted by activated cell death functions in kidneys in prediabetes may represent early events contributing to the known increased risk for steatosis/NASH and renal fibrosis/diabetic nephropathy comorbidities in people with type 2 diabetes. We further demonstrated that upregulated EV proteins and phosphoproteins involved in platelet activation, coagulation, chemokine signaling, and oxidative phosphorylation pathways are evident early during the development of diabetes.

## Figures and Tables

**Table 1:** Clinical characteristics of the study cohort.

	Healthy	PreT2D	T2D	*p*
*n*	10	10	10	
Sex = Male (%)	5 (50.0)	5 (50.0)	5 (50.0)	1.000
Age (years)	45.9 (10.5)	48.0 (7.8)	50.7 (11.0)	0.560
BMI (kg/m^2^)	33.1 (6.5)	34.1 (5.6)	34.7 (5.2)	0.825
Weight Average (kg)	89.7 (16.7)	101.0 (15.6)	97.8 (21.4)	0.368
Height Average (cm)	165.4 (7.8)	172.8 (11.1)	167.5 (9.6)	0.223
Waist Circumference (cm)	99.6 (16.4)	110.8 (15.8)	109.63 (16.2)	0.252
DEXA Lean Mass (g)	52,541.6 (8827.6)	56,993.2 (12,429.7)	54,111.7 (12,128.5)	0.672
DEXA Fat Mass (g)	34,737.9 (16,103.2)	41,785.8 (15,705.2)	41,624.5 (12,800.2)	0.494
DEXA Fat Percentage (%)	38.3 (14.0)	41.7 (12.2)	43.0 (9.3)	0.672
HDL-c (mg/dL)	55.9 (11.8)	42.2 (8.9)	48.7 (16.6)	0.076
LDL-c (mg/dL)	113.7 (26.5)	113.6 (46.7)	102.3 (37.0)	0.741
Triglycerides (mg/dL)	105.0 (60.4)	159.7 (134.1)	139.5 (53.4)	0.404
TSH (mIU/L)	1.6 (0.7)	2.1 (1.1)	2.4 (1.1)	0.180
Temperature (F)	98.0 (0.3)	97.9 (0.4)	97.8 (0.2)	0.605
Respiration Rate (breaths per min)	14.7 (2.1)	15.5 (1.8)	15.2 (1.9)	0.646
Systolic Blood Pressure (mmHg)	124.4 (10.1)	125.2 (10.4)	126.0 (11.9)	0.950
Diastolic Blood Pressure (mmHg)	78.30 (9.1)	77.2 (9.9)	79.80 (8.2)	0.815
Heart Rate (beats per min)	63.4 (8.4)	71.0 (11.5)	67.6 (13.4)	0.340
HbA1C (%)	5.4 [5.3, 5.5]	5.9 [5.8, 6.1]	6.3 [6.1, 7.0]	0.001
Glucose baseline (mg/dL)	90.3 (6.7)	100.6 (8.2)	122.2 (20.3)	<0.001
Insulin baseline (µIU/mL)	3.6 (2.2)	8.6 (6.8)	5.8 (5.8)	0.134
Glucose AUC (mg/dL∙min)	14,797.3 [13,302.1, 16,433.5]	17,202.3 [16,488.6, 18,909.1]	25,796.2 [23,196.5, 29,180.5]	<0.001
Insulin AUC (µIU/mL∙min)	3594.0 [2560.8, 7438.8]	6271.0 [2964.6, 11,626.3]	3463.6 [2254.2, 4915.9]	0.237
C-peptide AUC (ng/mL∙min)	694.2 [569.6, 820.0]	950.3 [573.5, 1262.8]	816.4 [687.6, 887.7]	0.783
Insulinogenic Index (ΔIns_0–30 min_/ΔGlu_0–30 min_)	0.9 (0.6)	0.3 (3.0)	0.2 (0.2)	0.623
HOMA-IR	0.8 (0.5)	2.1 (1.5)	2.0 (2.2)	0.155
HOMA_B	51.5 (36.4)	96.0 (107.6)	30.7 (22.5)	0.102
MATSUDA	12.2 (9.4)	6.3 (5.5)	9.7 (8.9)	0.276
AIRg	468.6 (349.8)	549.32 (530.1)	48.75 (64.6)	0.041
Si	4.6 (4.5)	2.7 (2.9)	4.6 (2.6)	0.434
DI	1150.7 (494.2)	890.3 (569.4)	249.5 (348.3)	0.005

Data presented as mean (standard deviation) or median [interquartile range]. Sex presented as number of males (%). Abbreviations: BMI: Body Mass Index, DEXA: dual-energy X-ray absorptiometry, HDL-c: high-density lipoprotein cholesterol, LDL-c: low-density lipoprotein cholesterol, TSH: thyroxin stimulating hormone, AUC: area under the curve, HbA1c: glycate hemoglobin, HOMA-IR: homeostasis model assessment insulin resistance, HOMA-B: homeostasis model assessment beta, AIRg: acute insulin response to glucose, Si: sensitivity index, DI: disposition index.

## Data Availability

Data supporting reported results can be found as supplementary material in Supplementary Data Tables SDT1 and SDT2.

## Supplementary Materials

The following supporting information can be downloaded at: https://www.mdpi.com/article/10.3390/ijms23105779/s1.
